# Simultaneous electroencephalography (EEG) and functional magnetic resonance imaging (fMRI) data during an inner speech task

**DOI:** 10.1016/j.dib.2025.112258

**Published:** 2025-11-12

**Authors:** Foteini Simistira Liwicki, Rajkumar Saini, Debashis Das Chakladar, Sumit Rakesh, Vibha Gupta, Marcus Liwicki, Johan Eriksson

**Affiliations:** a Luleå University of Technology, Department of Computer Science, Electrical and Space Engineering, Embedded Intelligent Systems LAB, Lulea, Sweden; b Umea University, Department of Psychology and Umeå center for Functional Brain Imaging, Umea, Sweden; c University of Gothenburg, Gothenburg, Sweden

**Keywords:** Multimodal neuroimaging, Inner speech, Synchronous data, Fmri, EEG

## Abstract

Inner speech, or covert speech, refers to the internal generation of language without overt articulation. Decoding inner speech has significant implications for brain-computer interfaces (BCIs), particularly for assistive communication in individuals with speech and motor impairments. To facilitate research in this area, we introduce a publicly available dataset comprising simultaneously recorded electroencephalography (EEG) and functional magnetic resonance imaging (fMRI) data during inner speech production.

Data were collected from three healthy, right-handed participants performing an inner speech task. The task involved silent repetition of visually presented words belonging to either a social or numerical category. The experiment consisted of 40 trials per word, with eight unique words and starts with a fixation period of two seconds. Stimuli were displayed for two seconds at the beginning of each session, followed by a 12-second rest period to allow hemodynamic responses to return to baseline. Participants were instructed to remain still and avoid movements to minimize artifacts.

EEG was recorded using a 64-channel MR-compatible cap (BrainCap MR, EasyCap GmbH) at a 5 kHz sampling rate. Electrocardiogram (ECG) signals were simultaneously acquired using an additional electrode placed on the trapezius muscle to facilitate cardioballistic artifact correction. Gradient and cardioballistic artifacts were corrected using BrainVision Analyzer software.

Functional MRI data were acquired using a 3T scanner with a 48-channel headcoil, and an echo-planar imaging (EPI) sequence optimized for whole-brain coverage. The repetition time (TR) was 2 s. High-resolution anatomical T1-weighted images were also acquired for structural reference. The dataset is publicly available in the OpenNeuro repository.

The aim of this dataset is to provide a resource for studying inner speech processing, multimodal neuroimaging, EEG-fMRI fusion techniques, and BCI-driven speech prosthesis development.

Specifications Table

**Table:** 

Subject	Health Sciences, Medical Sciences & Pharmacology
Specific subject area	Neuroimaging investigation of the human brain during rest and inner speech
Type of data	Preprocessed
Data collection	Simultaneous EEG and fMRI data were recorded following an inner speech task protocol from 3 righthanded healthy participants. The experimental protocol consists of 2 sessions with 2 s inner speech task period followed by a 12 s rest period. The EEG data were acquired using a 64-channel MR-compatible cap (BrainCap MR, EasyCap GmbH) at a 5 kHz sampling rate. The functional MRI data were acquired using a 3T scanner with a 48-channel headcoil, and an echo-planar imaging (EPI) sequence optimized for whole-brain coverage. High-resolution anatomical T1-weighted images were also acquired for structural reference.
Data source location	Data were collected: Umeå Center for Functional Brain Imaging (UFBI), Sweden Data are stored: Luleå University of Technology, Department of Computer Science, Electrical and Space Engineering, Sweden
Data accessibility	Repository name: OpenNeuro Data identification number: doi:10.18112/openneuro.ds006033.v1.0.0 Direct URL to data: https://openneuro.org/datasets/ds006033
Related research article	none

## 1 Value of the Data

• This dataset provides simultaneously recorded EEG and fMRI data during inner speech, a crucial yet underexplored cognitive process. The combination of these modalities allows researchers to investigate the neural correlates of inner speech with greater detail than unimodal datasets. fMRI data can be used to identify functional connectivity patterns, while EEG data can provide insights into oscillatory dynamics and event-related potentials supporting research in self-referential thought, language processing, and internal dialogue.
• The dataset serves as a benchmark for developing and validating EEG-fMRI fusion techniques. Researchers can test and compare methods for integrating EEG and fMRI data, assessing different fusion strategies, such as model-based approaches, data-driven techniques, and machine learning algorithms for feature selection.
• The data can support the development of BCIs for speech prostheses and silent communication. It also has clinical translation potential, particularly for designing assistive technologies for individuals with communication impairments, such as those with amyotrophic lateral sclerosis (ALS) or neurodevelopmental conditions including autism or ADHD.
• The dataset can contribute to studies on consciousness, particularly in relation to spontaneous mental activity, internal verbalization, and the dynamic engagement of the default mode network (DMN) during inner speech.
• Researchers can use the dataset to design and train machine learning models for inner speech recognition.

## 2 Background

Inner speech, or covert speech, refers to the internal generation of language without overt articulation. Understanding and decoding inner speech have significant implications for brain-computer interfaces (BCIs), particularly in developing assistive communication technologies for individuals with severe speech and motor impairments. Despite its potential, inner speech recognition remains a challenge due to the absence of articulatory movements and the complex neural mechanisms involved. Existing datasets often rely on unimodal neuroimaging [1], limiting the ability to fully capture the neural dynamics of inner speech.

This study introduces the first publicly available dataset with synchronously recorded electroencephalography (EEG) and functional magnetic resonance imaging (fMRI) data during inner speech and compliments our asynchronous bimodal dataset of inner speech following the same experimental protocol [2]. EEG provides high temporal resolution, enabling the capture of rapid neural activity, while fMRI offers superior spatial resolution, facilitating the localization of brain regions involved in inner speech processing. By integrating these complementary modalities, this dataset aims to support research on multimodal approaches for inner speech decoding [3].

## 3 Data Description

The preprocessed anonymized EEG and fMRI data are publicly available under the Creative Commons CC0 license in Brain Imaging Data Structure (BIDS) format (https://bids-specification.readthedocs.io/en/stable/) [4] at the OpenNeuro repository. The dataset includes simultaneously acquired EEG and fMRI data from 3 healthy participants. The data for each participant were recorded simultaneously using EEG and fMRI, and are organized into two sessions (ses-01 and ses-02), with data from each modality (EEG, fMRI) stored in separate BIDS-compliant folders. Each of the 8-word stimuli was assessed with 20 trials per session, resulting in 320 trials in each modality for each subject. There are 3 directories, one for each subject (sub-01, sub-02, and sub-03).

The anatomical MRI data are located in the anat folder and consists of the anatomical MR scan with file names in the following format: sub-XX_ses-YY-T1w, where XX denotes the subject ID and YY denotes the session ID. The functional data are available in the func folder, where the .nifti file and its corresponding .json file have the following format: sub-XX_ses-YY_task-innerspeech-bold, where XX denotes the subject ID and YY denotes the session ID. The task event file is also available as a .tsv file named sub-XX_ses-YY_task-innerspeech-events, where XX denotes the subject ID and YY denotes the session ID in the corresponding func folders. Note that the .nifti file includes the 10 dummy volumes that were acquired in the start of each session.

All EEG related files are stored in the corresponding eeg folder, organized per subject and session. The EEG data was collected synchronously during the fMRI scanning sessions using the BrainVision Recorder, Version 1.25.0204, Brain Products, Gilching, Germany. Each .eeg file is accompanied by the corresponding .json file, Brain Vision header file .vhdr and Brain Vision marker file .vmrk. The task event file is also available as a .tsv file named sub-XX_ses-YY_task-innerspeech-events, where XX denotes the subject ID and YY denotes the session ID in the corresponding func folders. Note that the data from sub-01, ses-01 were not recorded due to a technical issue and thus are not available. Note that the .eeg file does not include the recorded time of the 10 dummy volumes.

Note that the EEG dataset shared on OpenNeuro includes recordings that have been preprocessed to remove MRI gradient artefacts and cardioballistic artefacts using average artefact subtraction (AAS). R-peak events were extracted from the ECG channel after gradient artefact correction and are included in the .vmrk files. Raw, unprocessed EEG data are not included in the public repository but can be made available upon reasonable request.

## 4 Experimental Design, Materials and Methods

The bimodal dataset consists of synchronous EEG and fMRI data recorded during the performance of an inner speech task.

### 4.1 Participants

Three right-handed healthy participants participated in the experiment: one female and two males aged 25–35 years. None of the participants were native English speakers.

### 4.2 Experimental design

The experimental paradigm began with a 2-second fixation cross, after which each word trial consisted of a 2-second inner speech task period followed by a 12-secondrest period. This sequence was repeated 20 times per condition. During the task period, a word was presented visually on the screen, and participants were instructed to use their inner speech to repeat the word once. EEG and fMRI data were recorded simultaneously, using the same timing protocol across both modalities.

The stimuli comprised 8 words from 2 semantic categories (social and numerical), as described in [2]. The social category includes the following words: “daughter”, “wife”, “father”, “child”; the numerical category includes the words “three”, “four”, “six”, and “ten”.

Participants were instructed to engage in **inner speech**, defined as the internal production of the presented word without overt articulation or imagined auditory input. The standardized instruction was: *“When the word appears on the screen, please repeat it once silently in your mind, as if you were saying it to yourself, without moving your lips or tongue.”* This instruction is consistent with the definition of inner speech in [1] and was intended to promote consistent internal verbalization across trials.

### 4.3 Material

Both EEG and fMRI data were collected in a synchronous way with simultaneous EEG and fMRI recordings, following the experimental paradigm described in the previous section using the BrainVision Recorder software BrainVision Recorder, Version 1.25.0204, Brain Products, Gilching, Germany, to collect the EEG signals. The fMRI data were collected using 3T Signa Premiere MRI system from General Electric, equipped with a 48-channel head coil. Visual stimuli were presented on a display screen located at the head side of the scanner (GE 3T viewing angles: horizontal 23 deg., vertical 13 deg.).

### 4.4 EEG and fMRI acquisition

Before the bimodal acquisition, an MR-compatible EEG cap (BrainCap MR, EasyCap GmbH, Herrsching, Germany) was fitted onto the participant's head, with 64 electrodes positioned according to the 10–20 system. An appropriate cap size was selected for each participant by measuring the head circumference from nasion to inion, ensuring that the cap was properly centred with the Cz (Vertex) at the centre of the head, namely, halfway between the nasion and inion and halfway between the two ears. An electrolyte gel (ABRALYT 2000, EASYCAP GmbH, Herrsching, Germany) was applied between the electrodes and the scalp to provide electrode connectivity and minimize the impedance of the electrodes. The impedance of the electrodes remained below 60 kΩ for the majority of the channels, though a few channels exceeded this threshold likely due to anatomical or contact-related challenges. While this exceeds the < 50 kΩ threshold recommended for optimal EEG–fMRI quality [5], signal quality was deemed acceptable and MRI-related artefacts were corrected (see below). The MR cooling system was switched on during acquisition [6].

In addition to EEG recordings, an electrocardiogram (ECG) signal was recorded using an additional electrode attached to the distal part of the trapezius muscle. The EEG and ECG data were acquired using the Brain Vision Recorder (Brain Products, Gilching, Germany) while the participant lay in the scanner. During the recording, participants were instructed to remain still and relaxed. The EEG data were sampled at 5 kHz, with a bandwidth of 0.016–250 Hz. Finally, the raw EEG data underwent correction for gradient artifacts induced by the magnetic field and cardioballistic artifacts using BrainVision Analyzer, Version 2.3.0, Brain Products, Gilching, Germany.

Anatomical images were acquired using a sagittal T1-weighted 3D magnetization-prepared rapid acquisition gradient echo (MPRAGE) sequence with the following parameters: repetition time (TR) = 2567 ms; echo time (TE) = 2.84 ms; inversion time (TI) = 1 ms; flip angle = 8 degrees; number of slices = 364; FOV = 240 mm; matrix size = 240×240×364; and voxel size = 1 × 1 × 0,5 mm.

Structural images were acquired using an axial T2-Weighted Fast Spin Echo (Ax T2 FSE) sequence with the following parameters: TR=4.642 ms; TE= 90 ms; flip angle = 142 degrees; number of slices= 52; slice thickness = 3 mm; FOV=240 mm; matrix size=340×300×52.

B0 field map was obtained using gradient echo-planar imaging sequence with the following parameters: TR=8 ms; flip angle = 10 degrees; number of slices = 46; slice thickness = 4 mm; FOV= 256 mm; matrix size 64×64×46.

Functional MRI was obtained using an echo-planar imaging sequence with the following parameters: TR = 2.00 s; TE = 30 ms; flip angle = 80 degrees; slice thickness = 3.4 mm with a 0.5 mm gap between slices; number of slices = 37; FOV = 250 mm; matrix size = 96×96×37; voxel size = 2 × 2 × 3.4 mm. Note that every session starts with the acquisition of 10 dummy scans.

### 4.5 EEG artefact correction

EEG data recorded simultaneously with fMRI were corrected for scanner-related artefacts using BrainVision Analyzer 2.3. Gradient artefacts were removed using average artefact subtraction (AAS) with a 21-volume sliding template (TR = 2000 ms), with automatic detection of scanner triggers. The number of detected artefacts were equal to the number of acquired volumes, indicating that the detection of artefacts was succesfull. Baseline correction was applied using the full artefact interval, and artefacts with low correlation or signal quality were marked. All EEG channels were included in the correction, and no downsampling was applied.

Cardioballistic artefacts were subsequently corrected using AAS time-locked to R-peaks detected from the ECG channel. R-peaks were identified using a **semi-automatic detection algorithm**, with a pulse rate range of 48–80 beats per minute and a pulse duration window of 1000 ± 250 ms. The detection algorithm used a coherence threshold of 0.6 and amplitude constraints (0.6–1.2 a.u.). Detected R-peaks were marked and used to perform AAS with a **21-heartbeat sliding template**, and **automatic time-delay estimation** was applied. Correction was applied to 26 EEG channels spanning frontal, central, parietal, occipital, and temporal regions.

## Limitations

Limited number of participants, absence of EEG recordings for subject 01, session 01 due to technical reason, interrupted recording session of subject 02, session 01.

## Ethics Statement

All participants filled out an fMRI pre-screening form in order to exclude people that should not undergo the experimental procedure (due to the presence of metallic objects in their body, claustrophobia etc.) and signed the relevant informed written consent before the beginning of the experiment. The study was approved by the Swedish Ethical Review Authority (Etikprövning myndigheden, ID: 2023–05177–01) (https://etikprovningsmyndigheten.se/) in accordance with the Swedish Ethical Review Act (SFS 2003:460).

## CRediT Author Statement

**Foteini Simistira Liwicki:** project conception, methodology, experimental design, data acquisition, data curation, writing, and finalizing the manuscript, funding acquisition, project administration; **Rajkumar Saini:** data acquisition, data curation, writing, and reviewing the manuscript; **Debashis Das Chackladar:** data acquisition, data curation, writing and reviewing the manuscript; Sumit Rakesh: data acquisition, data curation, reviewing the manuscript; **Vibha Gupta:** data acquisition, writing and reviewing the manuscript; **Marcus Liwicki:** contributing in writing of the manuscript; **Johan Eriksson:** advised the experimental design, data acquisition, data curation and writing of the manuscript.

## Data Availability

OpenNeuroSynchronous EEG and fMRI dataset on inner speech (Original data).
